# Continuous glucose monitoring in the treatment of obesity in patients with glycogen storage disease type Ia

**DOI:** 10.1530/EDM-13-0056

**Published:** 2013-12-01

**Authors:** Betty Korljan Jelaska, Sanja Baršić Ostojić, Nina Berović, Višnja Kokić

**Affiliations:** 1 Department of Internal MedicineUniversity Hospital SplitSplitCroatia; 2 Department of RadiologyGeneral Hospital Sveti DuhZagrebCroatia

## Abstract

Glycogen storage disease (GSD) type I is characterized by impaired production of glucose from glycogenolysis and gluconeogenesis resulting in severe hypoglycaemia and increased production of lactic acid, triglyceride and uric acid. The most common type, glycogenosis type Ia, demands a balanced, sufficient carbohydrate intake to preserve normal 24-h glycaemia. Insufficient intake of carbohydrates can cause hypoglycaemia, as the missing glucose-6-phosphatase enzyme cannot free the glucose stored as liver glycogen and nor is gluconeogenesis possible. The principle means of handling this disorder is to avoid starving by taking regular meals during the day and night. Such a dietary regimen could lead to obesity. Herein, we present the case of an adult patient with glycogenosis type Ia suffering from hyperuricaemia, dyslipidaemia and arterial hypertension. The accumulation of these cardiovascular risk factors could lead to the early onset of atherosclerosis, which should be postponed by contemporary methods of surveillance and treatment.

**Learning points:**

- Continuous subcutaneous glucose monitoring may be of value in every adult patient with GSD type I to evaluate the actual prevalence of eventual hypoglycaemic and hyperglycaemic episodes.
- Good dietary management minimizes the metabolic abnormalities of the disease and decreases the risk of long-term complications.
- Treatment of obesity in patients with GSD reduces the risk of earlier atherosclerosis and cardiovascular disease.

## Background

Glycogen storage disease (GSD) type Ia (von Gierke's disease) is an inherited (autosomal recessive transmission) metabolic disorder of glycogen metabolism, caused by the deficiency of glucose-6-phosphatase (G6P) (1). G6P is an enzyme that is essential for providing glucose during fasting and is found mainly in the liver and kidneys. Both glycogenolysis and gluconeogenesis are affected. Initial symptoms and clinical signs occur in early childhood, usually during the first year of life. Patients present with protuberant abdomen due to hepatomegaly, relatively thin extremities, hypoglycaemia, lactic acidosis, hyperlipidaemia and hyperuricaemia. Trivial events (a short delay in taking a meal or a lower intake of carbohydrates as a consequence of an underlying illness) may elicit hypoglycaemia. The hypoglycaemic episodes typically do not respond to glucagon administration. Older infants present with a doll-like facial appearance, growth retardation and rachitic changes. Osteoporosis, renal function impairment, hepatic adenomas and even brain damage, probably caused by recurrent severe hypoglycaemia, may appear later in life. Patients with type Ib disease frequently suffer from bacterial infections caused by neutropaenia and have a tendency to nosebleed due to impaired platelet function.

The main goal of treatment is to preserve normal glucose levels and prevent hypoglycaemia, which normalizes the levels of triglyceride (TG) at the same time. Treatment includes frequent high-carbohydrate meals with slow-release glucose preparations, such as uncooked cornstarch, used during daytime to prolong the fasting period (2). In children, continuous nocturnal gastric drip feeding via a nasogastric tube has shown optimal results in maintaining normal glucose levels, allowing the patient and parents to sleep during the night. Good dietary management minimizes the metabolic abnormalities of the disease and decreases the risk of long-term complications (3). Patients are encouraged to control hypoglycaemia by standard glucose self-monitoring, usually preprandial. The fear of hypoglycaemia is often present, so patients tend to eat more and exceed their metabolic needs. The result is obesity, which contributes to the risk of earlier atherosclerosis and cardiovascular disease.

## Case presentation

**Figure 1:**
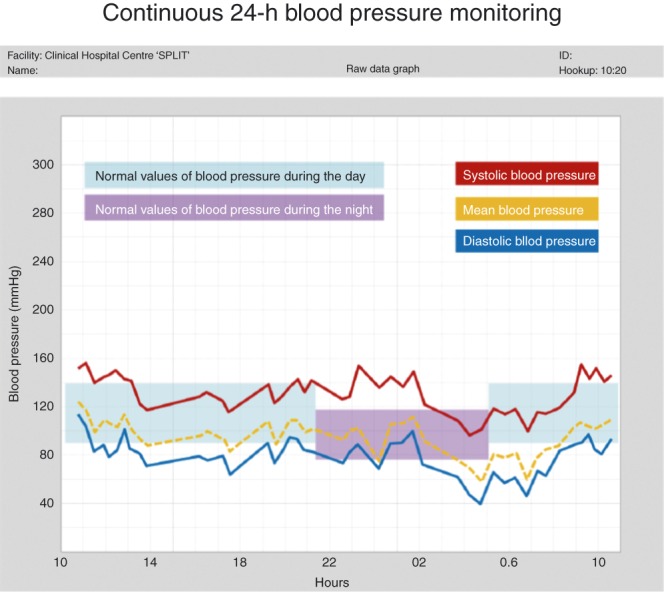
(a, b and c) Twenty-four-hour ambulatory blood pressure monitoring showing normal daily and average 24-h values of both systolic and diastolic pressure and the non-dipping pattern of the systolic pressure during the night.

We present the case of a 23-year-old female who was diagnosed with GSD type Ia shortly after birth. She was hospitalized for the first time at the age of 6 months for hypoglycaemia and metabolic acidosis that occurred during a respiratory infection. She had a doll-like facial appearance, hepatomegaly and kidney enlargement. She had been repeatedly hospitalized for episodes of enormous hypoglycaemia during infancy and adolescence. The patient was treated out of hospital with frequent meals rich in carbohydrates during the day and every 2–3 h during the night and as a result she developed obesity. Besides nutritional therapy, she was also treated with allopurinol for hyperuricaemia. She came to our clinic at the age of 22 years for the diagnostic evaluation of arterial hypertension and dyslipidaemia. Her weight was initially 80 kg, height was 157 cm and BMI was 32.5. The levels of uric acid were 425 nmol/l, LDL 3 mmol/l, HDL 0.8 mmol/l, cholesterol 5.2 mmol/l and TG 2 mmol/l. Her in-office blood pressure measurement repeatedly showed raised values (160/90 mmHg in average). We carried out a 24-h ambulatory blood pressure monitoring, which revealed normal daily and average 24-h values of both systolic and diastolic pressure, but also the non-dipping pattern of systolic pressure during the night (Fig. 1a, b and c). The latter can be attributed to excessive nocturnal eating and consequently disturbed sleeping pattern.

The patient used to control glycaemia with self-measurements, mostly in preprandial periods, and results were recorded in a blood glucose diary. The self-measurements were made only during the daytime and revealed no abnormal excursions of glucose levels in either direction.

It has been shown previously that glucose levels obtained by continuous subcutaneous glucose monitoring (CGM) correspond to those obtained by venous blood sampling or self-measurements (4) (5). To evaluate the actual prevalence of eventual hypoglycaemic episodes, we proposed CGM during a 72-h period.

## Investigation

**Figure 2:**
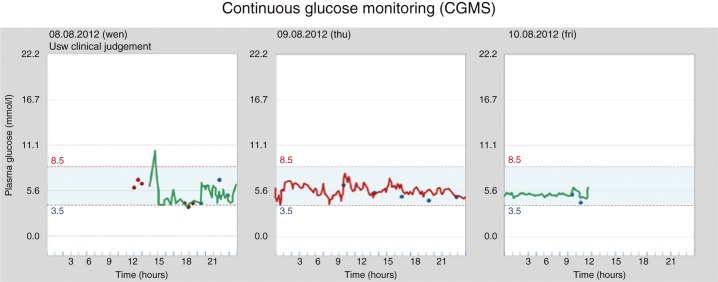
(a) One episode of postprandial hyperglycaemia recorded during the first day and (b and c) no episode of hypoglycaemia.

Glucose levels <3 mmol/l with symptoms that are relieved promptly when glucose levels are raised document hypoglycaemia (6). The results of CGM revealed no episodes of hypoglycaemia (the sensor was calibrated to the glucose range between 3.5 and 8.5 mmol/l). Instead, several episodes of postprandial hyperglycaemia were recorded (Fig. 2a, b and c).

## Treatment

The patient was advised by the nutritionist to change her dietary regimens. She reduced the quality and quantity of meals and cut out the candy syrup that she was using frequently for many years because of the fear of hypoglycaemia. She was advised to use cornstarch instead.

She used 400 g of cornstarch initially and only 250 g/day later on.

## Outcome and follow-up

The patient came to our clinic at the age of 22 years for the diagnostic evaluation of arterial hypertension and dyslipidaemia. Her BMI was 32.5 at the time. The results of 72-h CGM revealed fluctuations in her blood glucose levels and allowed us to advise her to change her dietary regimens. She reduced the number and quantity of meals and lost 11 kg with a consequent decrease in BMI from 32.5 to 28 within a year.

Her lipid status also improved with an increase in the levels of HDL (1.2 mmol/l) and a decrease in the levels of TG (1.8 mmol/l), cholesterol (5 mmol/l), LDL (2.8 mmol/l) and uric acid (366 nmol/l).

## Discussion

To our knowledge, CGM revealing hypoglycaemic episodes in adult patients suffering from GSD has not been described so far. One study has found it useful in the assessment of nocturnal hypoglycaemia in children with GSD, while its usefulness in monitoring children with type I diabetes is well known (7) (8) (9). Most of the episodes of nocturnal hypoglycaemia are asymptomatic and not detected because patients do not perform glucose self-monitoring during the night. CGM offers an opportunity to capture data for retrospective analysis and to alert patients when blood glucose levels are low. It can also facilitate the detection of all postprandial glucose peaks. Patients with GSD are prone to developing obesity because of the constant fear of hypoglycaemic events. The long-standing moderate-to-severe dyslipidaemia may suggest early atherosclerosis, but there is a surprising lack of signs of vascular damage in these patients, probably because of the raised apoE levels in the serum, counterbalancing the increased risk of atherosclerosis (10). Nevertheless, the fact that several risk factors for developing cardiovascular disease are present in such patients should not be ignored. We suggest that CGM can be advantageous to glycogenosis type I patients for self-monitoring their glucose levels. CGM for a period of 72 h provides better insights into glycaemic fluctuation. It relieves the fear of frequent hypoglycaemia and diminishes excessive food intake, decreasing obesity and co-morbidity. It is not possible to recommend that CGM replace blood glucose self-monitoring, but we suggest that intermittent application of CGM can be used as a fine tool to detect nocturnal hypoglycaemia and, in this case, episodes of hyperglycaemia and help patients with GSD modify their dietary regimens.

## Patient's perspective

After knowing the results of CGM, I significantly reduced the use of cornstarch and thus lost weight and decreased the BMI.

## Patient consent

Consent was obtained from the patient for the publication of the case report.

## Author contribution statement

Betty Korljan Jelaska, MD, PhD, supervised and treated the patient and was responsible for the conception of case report, hypothesis and patient selection. Sanja Baršić Ostojić, MD, participated in analysis, data interpretation and case report writing. Nina Berović, MD, participated in the search of pre-existing literature, writing of the case report and critical revision and final editing of the paper. Višnja Kokić, MD, participated in pre-existing literature search, data acquisition from the patient and case report writing.
